# Simulating Pulp Vitality Measurements via Digital Optical Twins: Influence of Dental Components on Spectral Transmission

**DOI:** 10.3390/s25103217

**Published:** 2025-05-20

**Authors:** David Hevisov, Thomas Peter Ertl, Alwin Kienle

**Affiliations:** 1 Institute for Laser Technologies in Medicine and Metrology at the University of Ulm, 89081 Ulm, Germany; 2 DeguDent GmbH, Dentsply Sirona, 63457 Hanau, Germany

**Keywords:** digital optical twin, pulp vitality assessment, Monte Carlo simulation, anisotropic light propagation

## Abstract

Optical diagnostic techniques represent an attractive complement to conventional pulp vitality tests, as they can provide direct information about the vascular status of the pulp. However, the complex, multi-layered structure of a tooth significantly influences the detected signal and, ultimately, the diagnostic decision. Despite this, the impact of the various dental components on light propagation within the tooth, particularly in the context of diagnostic applications, remains insufficiently studied. To help bridge this gap and potentially enhance diagnostic accuracy, this study employs digital optical twins based on the Monte Carlo method. Using incisor and molar models as examples, the influence of tooth and pulp geometry, blood concentration, and pulp composition, such as the possible presence of pus, on spectrally resolved transmission signals is demonstrated. Furthermore, it is shown that gingival blood absorption can significantly overlay the pulpal measurement signal, posing a substantial risk of misdiagnosis. Strategies such as shifting the illumination and detection axes, as well as time-gated detection, are explored as potential approaches to suppress interfering signals, particularly those originating from the gingiva.

## 1. Introduction

Accurate assessment of pulpal health is essential in endodontic diagnosis and treatment planning, as it directly influences the clinical decision on whether and which endodontic treatment is required. Currently, the evaluation of pulp vitality relies on a multifactorial diagnostic process, integrating information from the patient’s medical history and chief complaint, as well as clinical and radiographic examinations, in conjunction with pulp sensibility tests [1]. The latter are based on the patient’s subjective perception of external stimuli, such as thermal or electrical excitation, and the clinician’s interpretation of the response [2]. However, the inherent subjectivity of these conventional methods, coupled with the fact that they assess only neural responses, without directly reflecting the vascular status of the pulp, introduces a considerable risk of false-positive and false-negative results [3], thereby posing a significant diagnostic challenge. Furthermore, sensibility testing is often associated with discomfort or even pain for the patient [4]. Given these limitations, there is a pressing need for objective, non-invasive, and pain-free diagnostic modalities to enhance the reliability of pulp vitality assessments. In response, research into optical diagnostic techniques based on reflected or transmitted light measurements has gained considerable momentum. Notably, pulse oximetry (PO) [5] and laser Doppler flowmetry (LDF) [6,7] have emerged as promising approaches for directly assessing pulp vitality by detecting blood oxygen saturation or intrapulpal blood flow, respectively, while ongoing research continues to explore several other methods [8]. Due to various limitations [8,9,10], such as high cost, complex calibration, operator dependence, influence from peripheral tissue, and the lack of universal standardization, ‘direct’ diagnostic methods have yet to achieve widespread clinical adoption and remain largely confined to the research sector. Recently, another simple optical approach, optical pulp scanning (OPS) [11], was introduced. This method is based on the acquisition of spectrally resolved transmission measurements, utilizing spectral features, such as blood absorption, to classify teeth into different vitality categories. However, diagnostic accuracy has so far remained limited.

To enable future advancements in optical diagnostic techniques and address existing diagnostic limitations, it is essential to first establish a fundamental understanding of light propagation within the tooth, as this ultimately determines the diagnostic output. In particular, it is crucial to investigate how various factors, such as tooth and pulp geometry and oxygen saturation and concentration, as well as the material composition of the pulp, affect light propagation. Since such an analysis is challenging to control experimentally, this study employs digital optical twins, which offer the significant advantage of allowing different model components to be systematically varied and independently analyzed. Therefore, a tetrahedron-based GPU (graphics processing unit)-accelerated Monte Carlo (MC) simulation software, explicitly accounting for anisotropic light propagation in dentin, was developed to investigate the dynamics of various model parameters in the context of spectral transmission measurements, based on multi-volume 3D datasets of an incisor and a molar. Specifically, the influence of different pulp compositions, oxyhemoglobin, deoxyhemoglobin, and pus, as well as varying blood concentrations on the transmission signal and internal light distribution was examined, to elucidate in detail the underlying mechanisms by which changes in the scattering and absorption properties of the pulp alter the spectral information, particularly the visibility of blood absorption features. In addition to transmission spectra, the impact of the gingiva was quantified by tracking photon path lengths. The results indicate that spectral features are significantly influenced by tooth geometry and gingival absorption, ultimately compromising the diagnostic accuracy of such an approach. Furthermore, various strategies, such as adjustments to the illumination and measurement positions, as well as time-gating, were evaluated to determine their effectiveness in mitigating these confounding factors.

## 2. Materials and Methods

### 2.1. Monte Carlo Model

**Figure 1:**
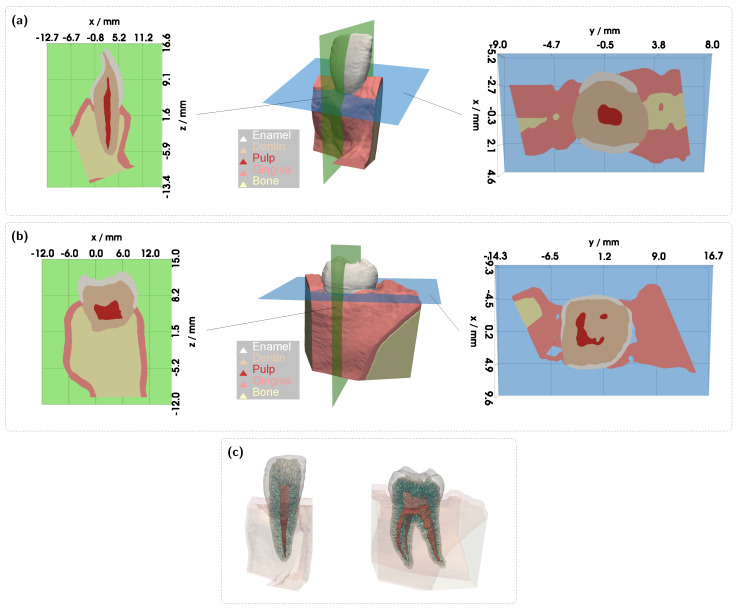
Overview of the digital optical twins. (**a**) A 3D model of the incisor with vertical (green) and horizontal (blue) sections. (**b**) A 3D model of the molar with vertical (green) and horizontal (blue) sections. (**c**) Internal view of the tooth models with assumed dentinal tubule orientations.

Regarding light propagation, a human tooth can be anatomically categorized into three principal layers: enamel, dentin, and pulp (see Figure 1a,b). The outermost layer, enamel, is primarily composed of hydroxyapatite [12]. Due to its relatively low scattering properties, enamel contributes to the characteristic translucency of the tooth’s incisal edge. Beneath the enamel lies dentin, which constitutes the bulk of the tooth’s volume and, like enamel, consists predominantly of hydroxyapatite, along with collagen and water [12]. Embedded within the dentin are microscopic supply channels known as dentinal tubules, which contain odontoblast extensions, a serum-like fluid, and occasionally nerve fibers. These tubules function as conduits for external stimuli transmission and play a crucial role in dentin physiology. In addition to their physiological significance, dentinal tubules are primarily responsible for the high scattering properties observed in dentin. Moreover, the radial arrangement of these structures, extending from the pulp toward the enamel, induces anisotropic light propagation within the tooth [13,14]. This results in a light-guiding effect, facilitating the transmission of light into the tooth’s inner regions. Similar anisotropic scattering phenomena have been observed in other aligned fibrous tissues, such as muscle and skin, as well as in biological structures like wood. At the core of the tooth, the pulp consists of innervated and vascularized connective tissue, serving both nutritive and sensory functions.

The complex, multilayered 3D geometry of the tooth, combined with the anisotropic light propagation induced by dentinal tubules, necessitates a specialized simulation approach that extends beyond standard MC models. Therefore, all subsequent simulations were conducted using a custom-developed, tetrahedron-based, GPU-accelerated MC software. This enables the investigation of diagnostic questions using highly realistic digital optical twins of teeth. Furthermore, the tetrahedral representation of the simulation domain offers the advantage of accurately accommodating arbitrarily shaped 3D objects, such as teeth, ensuring a high degree of geometric accuracy. To create the simulation models, segmented cone beam computed tomography (CBCT) scans acquired in vivo from a healthy maxillary central incisor (tooth 21) and a mandibular first molar (tooth 36) were utilized (Clinic for Orthodontics, Ulm University Hospital). These scans were processed using TetGen to generate a tetrahedral mesh. In addition to the teeth, the corresponding alveolar bone, in which the teeth are embedded, as well as the overlying gingiva, were incorporated as separate volumetric domains to allow for their inclusion in the simulation model. Figure 1a,b provide an overview of the complete models (center), along with vertical (left) and horizontal (right) cross-sectional views. As previously mentioned, the approximately cylindrical microstructures within the dentin significantly influence light propagation. To accurately simulate the associated anisotropic light propagation effects, a model was employed that accounts for both the tubule-dependent scattering, which varies with the incident light direction relative to the tubules’ direction, and the isotropic light propagation occurring within the surrounding dentin matrix [15]. This approach requires knowledge of both the scattering efficiency and phase function for each angle of incidence. To this end, the solution of Maxwell’s equations for the scattering of a plane wave by an infinitely extended cylinder was applied [16]. The relevant scattering parameters were precomputed and stored in a wavelength-resolved lookup table (LUT) for incidence angles $\zeta \in [0^{\circ },90^{\circ }]$, with a step size of $0.5^{\circ }$. For all simulations, a tubule diameter of 1 μm [17] and a homogeneous area number density of 45,000 mm^−2^ [18] were assumed. Furthermore, within the dentin volume, each tetrahedral element was assigned a directional vector characterizing the orientation of the embedded tubules (see Figure 1c). The direction was determined by the orthogonal projection of each tetrahedral centroid onto the pulp surface. For the dentin matrix and all other tooth regions, the rotationally symmetric Henyey–Greenstein phase function [19] was employed, meaning that in these cases, the scattering phase function remained independent of the incident direction. Notably, neglecting the anisotropic propagation of light in dentin can lead to substantial deviations in simulation results and, particularly in transmission configurations, may cause the pulp filling to have little or no effect on the spectral signal [15,20]. For further details on the implementation of anisotropic light propagation, the authors refer to [15]. The full simulation framework was validated in prior work through comparisons between experimental and simulated data, including spatially resolved reflectance measurements on tooth sections, spectral transmission measurements on teeth, and, more broadly, via a transillumination setup applied to prepared teeth [20,21].

The remaining simulation routine largely follows the standard MC methodology, employing the weighting technique complemented by the Russian roulette algorithm for photon termination [22]. Starting from the light source, which is modeled as an optical fiber with a radius of r=250 μm and a numerical aperture of NA=0.2, a total of *N* independent energy packets are launched into the simulation domain, with the total injected power arbitrarily set to 1 W. Since these so-called photons, in MC terminology, do not interact with one another, the MC method is particularly well suited to massive parallelization using GPU hardware. During their propagation through the simulated object, photons undergo various interactions with the participating media, governed by the optical properties of the respective subvolume, including scattering, absorption, refraction, and reflection. To this end, random numbers are utilized to sample probability distributions derived from the underlying physical laws, such as Lambert–Beer’s law and the Fresnel equations, to generate these stochastic photon trajectories. Photons may eventually reach the detector, which is also modeled as an optical fiber with a radius of r=500 μm, where their current weight is saved to the results array. As N→∞, the simulation results formally converge toward the solution of the underlying physical model, the radiative transfer equation (RTE), which serves as an approximation of Maxwell’s equations. Due to its ability to provide a numerical solution to the RTE, the MC method is widely regarded as the gold standard in biophotonics for modeling light propagation in scattering media, particularly in cases where the geometric complexity or spatial extent of the simulation domain renders an analytical or numerical solution of Maxwell’s equations impossible. Furthermore, regarding the detector, the intensity is recorded in a time-resolved fashion. This is achieved by updating the cumulative photon travel time at each interaction point, i.e., scattering event, accounting for the speed of light specific to the current medium. Consequently, each photon is allocated to its corresponding temporal bin upon reaching the fiber-based detector. In addition to detecting transmitted signals, the time-resolved light distribution within the tooth model is also captured. To this end, the mean fluence within each tetrahedron is calculated efficiently based on scattering events occurring within the mesh element. Specifically, at each scattering event, the photon weight is divided by the product of the local scattering coefficient and the tetrahedral element’s volume, and subsequently accumulated in the corresponding temporal bin of the fluence detector. Further details regarding the implementation of the MC method and the solution of the RTE can be found in [23].

### 2.2. Optical Properties

**Figure 2:**
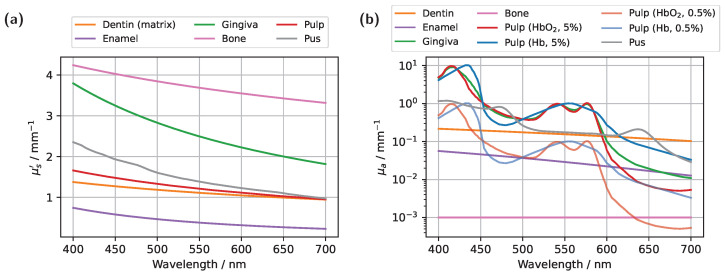
Overview of the assumed optical properties of the individual model components. (**a**) Effective scattering coefficients. (**b**) Absorption coefficients.

**Figure A1:**
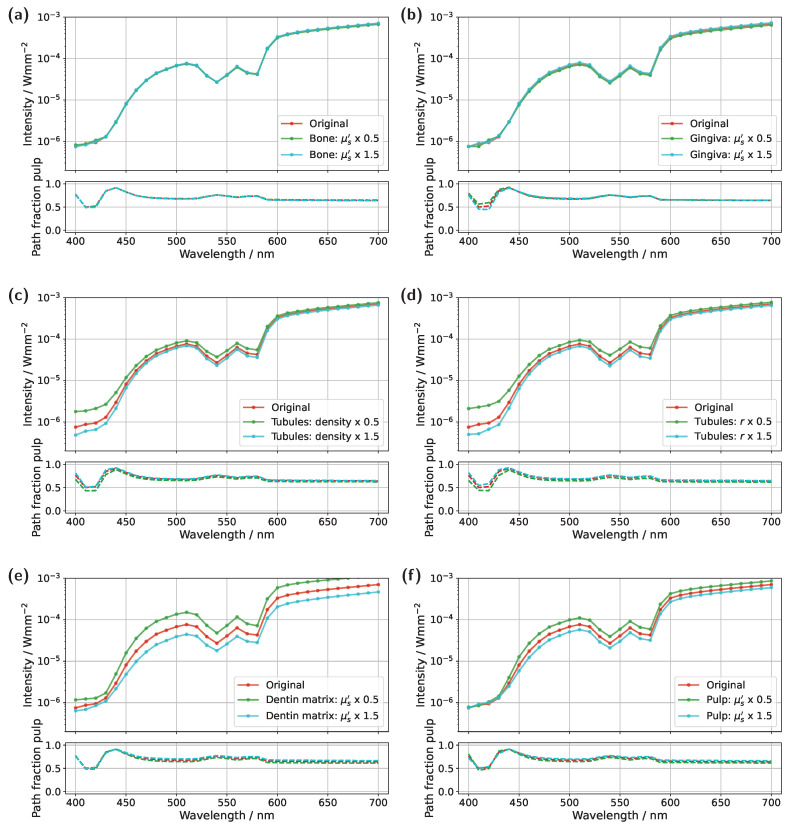
Simulated transmission spectra for the unmodified incisor model with a pulp filled with HbO_2_, under variation of the scattering properties of different model components. (**a**) Bone $\mu_{s}^{\prime }$. (**b**) Gingiva $\mu_{s}^{\prime }$. (**c**) Density of dentin tubules. (**d**) Radius of dentin tubules. (**e**) Dentin matrix $\mu_{s}^{\prime }$. (**f**) Pulp $\mu_{s}^{\prime }$. The respective subplots illustrate the pulp path fraction $s_{p}/\left(s_{p}+s_{g}\right)$ as a function of wavelength.

In the context of the radiative transfer theory, a medium is fully characterized by the absorption coefficient $\mu_{a}$, the scattering coefficient $\mu_{s}$, the refractive index *n*, and the scattering phase function $p(\hat{s},{\hat{s}}^{\prime })$. As their names imply, $\mu_{a}$ and $\mu_{s}$ quantify the probabilities of a photon being absorbed or scattered along a path segment, respectively. The refractive index, in turn, is used to determine transmission and reflection probabilities via the Fresnel equations, and it accounts for refraction at mismatched interfaces according to Snell’s law. Finally, the phase function describes the angular dependency of scattering, representing the probability that a photon traveling in direction $\hat{s}$ will scatter into direction ${\hat{s}}^{\prime }$. In biophotonics, the Henyey–Greenstein phase function is commonly employed, which is parameterized by the anisotropy factor *g*. For a realistic digital optical twin, it is therefore essential to know all these optical properties for each simulated volume, particularly enamel, dentin, and pulp, as well as gingiva and bone. However, determining the optical properties of teeth is challenging. Firstly, the complex 3D geometry combined with difficult in vivo measurement conditions makes direct tooth measurements impractical, thus often necessitating the use of ex vivo approaches and addressing their associated complications. Further complicating matters are inherent biological variability among individuals and additional influences related to lifestyle and health conditions, such as tooth discoloration or the presence of tertiary dentin. Nevertheless, arguably the most significant challenge is the anisotropic light propagation in dentin, caused by its tubular microstructure. If this anisotropy is not accurately represented in the underlying light propagation model, e.g., by assuming a single rotationally symmetric scattering function such as the HG phase function, substantial discrepancies from the actual light distribution can be expected [15,20]. Given these complexities, the optical properties employed in this study, derived primarily from data found in the literature [24,25,26,27,28,29], should be regarded as approximate reference values, primarily serving to qualitatively elucidate the influence of different model components within diagnostic scenarios. It should be noted that the optical properties of real teeth may vary by at least ±50% around these literature values. Although this implies that quantitative deviations from real transmission measurements in teeth are to be expected, it does not diminish the validity of the present simulations. The observed trends resulting from variations in different parameters are expected to be analogous in real teeth; however, the extent of these effects will depend on tooth-specific optical properties, geometry, and physiological parameters, varying from case to case. This is demonstrated in Figure A1 and Table A1, using the example of variations in the scattering properties of different model components. Figure 2 summarizes the absorption coefficients and effective scattering coefficients $\mu_{s}^{\prime }=\mu_{s}\left(1-g\right)$ used for all simulated volumes within the visible spectral range.

In the literature, various measurements consistently confirm that both the scattering and the absorption coefficient in dentin are significantly higher than in enamel [24,25,26,27]. This observation is also reflected in the assumed optical properties. However, it is important to note that in Figure 2a, the depicted scattering coefficient for dentin represents only the estimated scattering contribution of the intertubular component, i.e., the dentin matrix excluding the tubules. This estimation is based on values presented in [26], assuming an anisotropy factor of g=0.9. The calculations in [26] indicate that within the dentin matrix, collagen fibrils serve as the primary scatterers. The remaining, substantially larger contribution to dentin scattering [26], which arises from the tubules, is accounted for separately in this study through cylinder scattering. As previously mentioned, this tubular contribution is modeled using the solution of Maxwell’s equations for an infinitely extended cylindrical scatterer. In this case, the scattering coefficient and phase function are calculated based on the area number density of the tubules, assumed to be 45,000 mm^−2^ [18], with a diameter of 1 μm [17], and refractive indices of 1.35 (inner) and 1.54 (outer). Due to the anisotropic nature of the aligned scatterers, both quantities, the scattering coefficient and the phase function, exhibit a dependence on the incident angle relative to the cylinder axis. For the scattering properties of the pulp, reference was made to loose connective tissue [28,29,30], while the absorption coefficient $\mu_{a}$ was assumed to be primarily governed by blood. Blood volume fractions of 0.5% and 5.0% were examined to cover a wide range of possible values, with absorption properties derived from the absorption spectrum of whole blood [31]. The scattering and absorption properties of ‘human’ pus, which could not be found in the literature, were determined via integrating sphere measurements [32,33], assuming a refractive index of n=1.39 and an anisotropy factor of g=0.8. For bone, a high scattering coefficient coupled with minimal absorption was assumed, due to the scarcity of established values in the literature and the anticipated negligible optical influence within the simulation. In the case of gingiva, absorption was modeled based on a blood volume concentration of 5.0%, with an oxygen saturation of 80.0% [34,35], while the scattering properties were approximated using those of dense connective tissue [28,29,36]. The refractive index and anisotropy factor for the various tissues were adopted from previous studies, ranging from 1.39 to 1.63 for *n* and from 0.8 to 0.9 for *g* [15].

## 3. Results and Discussion

Blood absorption in the pulp provides information about the vascular status and, consequently, the vitality of the pulp. Ideally, in the case of a healthy pulp, approximated here by an oxygen saturation (SO_2_) of 100%, the characteristic HbO_2_ peaks at approximately 540 nm and 575 nm (see Figure 2b) would be prominently reflected in the detected transmission signal. In contrast, in a non-vital scenario, modeled in the extreme case as either 0% oxygen saturation or entirely filling with pus, these characteristic HbO_2_ peaks would be expected to disappear. This would provide a straightforward and objective approach for assessing pulp vitality. Beyond other features of the spectrally resolved transmission signal, such as the slope (first and second derivative), integrals under specific curve segments, and inflection points of the curves, this principle also formed the basis for classification in a recently proposed approach [11]. However, in that case, no clinically acceptable level of diagnostic confidence was achieved. To address this, digital optical twins will be utilized to gain a deeper understanding of light propagation within the tooth under similar measurement conditions, thereby investigating the underlying causes of insufficient diagnostic accuracy. All subsequent simulations were performed on an NVIDIA A10 GPU, with the computation time for generating a complete transmission spectrum averaging approximately one hour for $10^{9}$ photons.

### 3.1. Spectral Transmission of the Incisor Model

**Figure 3:**
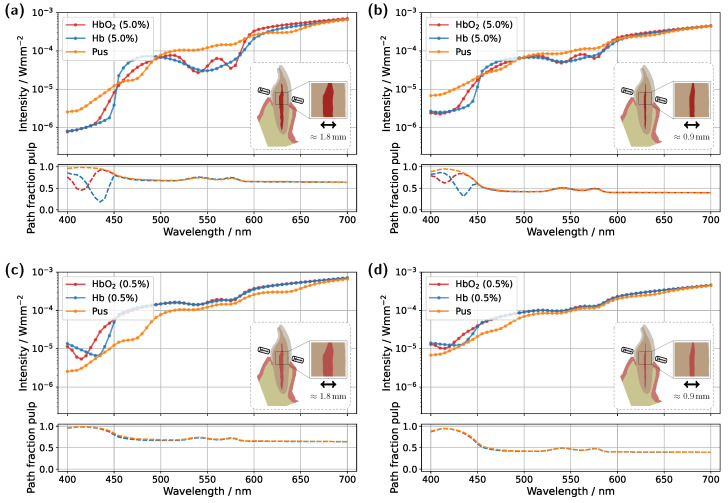
Simulated transmission spectra for the incisor model. (**a**) Unmodified tooth model with a blood concentration of 5.0% in the pulp. (**b**) Tooth model with a reduced pulp volume and a blood concentration of 5.0%. (**c**) Unmodified tooth model with a blood concentration of 0.5% in the pulp. (**d**) Tooth model with a reduced pulp volume and a blood concentration of 0.5%. The insets display vertical cross-sections of the tooth model, highlighting the maximum pulp extension along the section plane. The respective subplots illustrate the pulp path fraction $s_{p}/\left(s_{p}+s_{g}\right)$ as a function of wavelength.

Starting with the incisor model, transmission was compared for a detector and source positioned directly above the gingiva across different pulp sizes and blood concentrations (see Figure 3). The detector and source were aligned along an axis corresponding to the intersection of the two sectional planes indicated in Figure 1a. In all cases, the signal was also analyzed for a scenario in which the pulp chamber is completely filled with pus. In the lower subplots of each figure, the proportion of detected photons that traveled through the pulp relative to the gingiva is shown as $s_{p}/\left(s_{p}+s_{g}\right)$, where $s_{p}$ and $s_{g}$ denote the cumulative path lengths traveled within the pulp and gingiva, respectively.

**Figure 4:**
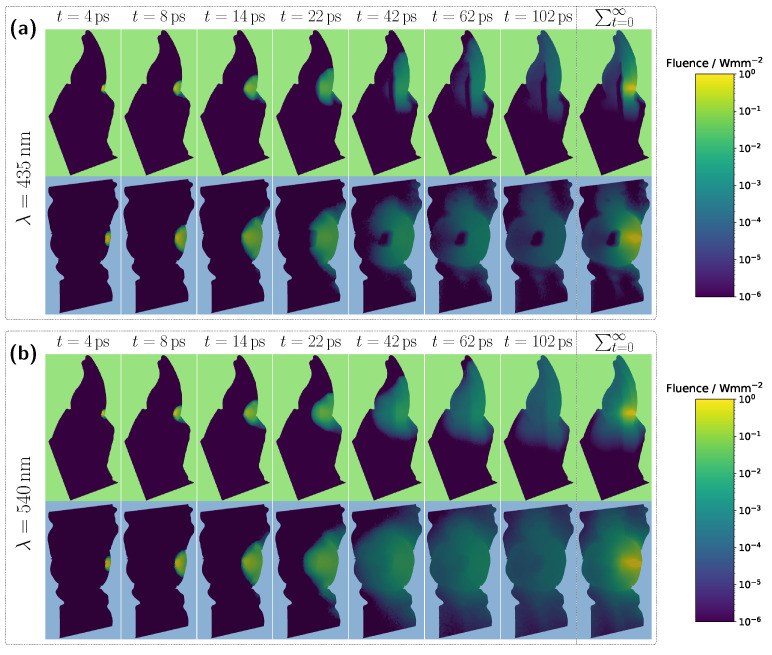
Time-resolved evolution of fluence for the unmodified incisor model with a pulp filled with deoxyhemoglobin at a blood concentration of 5.0%. (**a**) Incident wavelength of 435 nm. (**b**) Incident wavelength of 540 nm. The top and bottom rows show the vertical and horizontal cross-sections of the tooth model, respectively. The rightmost column in each case represents the steady-state distribution of fluence.

For the original model with a blood concentration of Hb/HbO_2_ at 5.0% (see Figure 3a), dips in the transmission spectrum are observed at the characteristic HbO_2_ absorption peaks (540 nm and 575 nm) in the case of 100% oxygen saturation, as expected. These features disappear in the Hb case, i.e., SO_2_ =0%. Surprisingly, however, even in the scenario where the pulp is completely filled with pus, slight but noticeable HbO_2_ absorption peaks remain visible, despite the assumption that no HbO_2_ is present within the pulp. This provides the first indication of the underlying cause of the diagnostic ambiguities, which can be inferred from the subplot in Figure 3a. In the wavelength range between 500 nm and 600 nm, the pulp path fraction decreases to approximately 70%, indicating that a significant portion of the detected photon paths traverse the gingiva. In the case of an Hb-filled pulp, due to the approximately equal blood concentration in both the gingiva and pulp, combined with the fact that the optical path length through the pulp is nearly twice as long as that through the gingiva, no features of HbO_2_ absorption from the gingiva are recognizable. Conversely, for a pus-filled pulp, gingival HbO_2_ absorption becomes distinctly visible, as blood absorption in the gingiva is significantly higher than that of the pus within the pulp. Additionally, the spectral profile of pus remains relatively constant in the region of the HbO_2_ dips (see Figure 2b), allowing the gingival HbO_2_ absorption to emerge more prominently. Notably, the visibility of the gingival HbO_2_ peaks increases with higher blood concentration and oxygen saturation in the gingiva and vice versa. As such, this effect may be influenced not only by biological variability but also by factors such as gingivitis [34]. Furthermore, it can be generally observed that high absorption in the pulp, such as that caused by the HbO_2_ and Hb peaks in the 400–450 nm range, directly corresponds to a reduction in the pulp path fraction, as reflected in the lower subplot of Figure 3a. This effect can be understood by analyzing the internal light distribution within the tooth, as represented by the simulated fluence in Figure 4.

Examining the time progression of the light distribution at a wavelength of 435 nm (see Figure 4a), where the absorption maximum of the Hb-filled pulp occurs in the visible spectral range, reveals that the pulp largely obstructs photon paths propagating in the incident direction. As a result, incoming light can barely penetrate the pulp and is predominantly absorbed within it, forcing photons to propagate around the pulp before reaching the detector. Consequently, detected light is more likely to interact with peripheral tissues, such as the gingiva, before being recorded. This effect is also clearly visible in the steady-state fluence distribution, shown in the rightmost column of Figure 4a. A different pattern emerges when analyzing the light distribution for an Hb-filled pulp at a higher wavelength of 540 nm (Figure 4b), where attenuation is significantly lower in both the pulp and surrounding tooth tissue, as seen in Figure 2. Although photons can now penetrate deeper into the surrounding tissue, the probability of reaching the detector decreases for these longer paths due to absorption and scattering. In contrast, photons traveling almost directly along the incident direction through the pulp are more likely to reach the detector, leading to an increased contribution of ‘direct’ photon paths and a higher pulp path fraction in the detected signal. Furthermore, regarding the pulp path fraction, it can be observed, particularly in the lower subplot of Figure 3a from approximately 450 nm onward, that when attenuation within the pulp is sufficiently low, the ratio between $s_{p}$ and $s_{g}$ is primarily dictated by the optical properties of the gingiva. For instance, in the region of the oxyhemoglobin double absorption peaks, the pulp path fraction increases due to enhanced photon absorption within the gingiva. It is worth noting that, in this case, while an absolute decrease in $s_{p}$ is also observed for an HbO_2_-filled pulp, the corresponding decrease in $s_{g}$ is markedly more pronounced. Another notable observation concerns the transmission curve for a pus-filled pulp. At approximately 475 nm and 640 nm, distinct absorption peaks corresponding to the measured pus spectrum, see Figure 2b, are visible, distinguishing the transmission spectrum from the blood-filled cases through these additional spectral features.

In the next step, the simulation model was adjusted to investigate the influence of a reduced blood concentration in the pulp on the transmission spectra (see Figure 3c). To model a non-vital pulp [37], the blood concentration for both Hb- and HbO_2_-filled pulp was set to 0.5%. As before, the third pulp condition was modeled as completely pus-filled. While a fully pus-filled pulp represents an extreme clinical scenario, given that such a condition would likely be accompanied by severe symptoms such as intense pain or swelling [38], this assumption is once again considered as a limiting case to better understand and illustrate the dynamics of the different model components. The reduction in absorption along the pulp due to the lower blood content leads to two key changes in the transmission spectra for oxygenated and deoxygenated blood-filled pulps. Firstly, the detected intensity in the transmission spectra for both of these cases is significantly higher in Figure 3c compared to the previous setup in Figure 3a, as less light is absorbed within the pulp. Secondly, distinct dips at approximately 540 nm and 575 nm now also appear in the transmission spectrum of the Hb case. From the lower subplot of Figure 3c, it is evident that a significant portion of the detected light has traversed the gingiva. While the pulp path fraction remains similarly high compared to the previous case, seen in Figure 3a, the absorption in the pulp is now approximately an order of magnitude smaller than in the gingiva, making the latter the dominant factor shaping the final signal. The strong influence of gingival absorption is further reinforced by the observation that, from approximately 450 nm onward, the transmission spectra for the HbO_2_- and Hb-filled pulps almost completely overlap, indicating that the pulp composition has only a minor effect on the spectral shape. In contrast, for wavelengths below 450 nm, the absorption maxima of oxygenated and deoxygenated hemoglobin now become visible in the transmission spectrum, opposite to the trend seen in Figure 3a. This is because the pulp no longer absorbs the majority of light in this spectral interval, whereas the gingiva does. The latter also explains why, in Figure 3c, the pulp path fractions already align at wavelengths below 450 nm. Based on the previously analyzed simulations, it becomes evident that the spectral analysis of transmission signals carries inherent risks of misinterpretation. The present example demonstrates that peripheral tissues, such as the gingiva, can significantly influence the detected transmission spectrum. In particular, blood absorption in the gingiva can overlay spectral characteristics, creating the false impression that oxygenated blood is still present in the non-vital pulp and thereby leading to an incorrect diagnosis. The authors note that in various other optical diagnostic methods, it has already been suspected that the gingiva may contribute to inconsistent results [8].

In addition to varying pulp composition and blood concentration, the pulp geometry was also adjusted in the simulations shown in Figure 3b,d to account for a reduction in pulp cavity size due to pathological conditions or natural age-related changes [39,40]. Specifically, the radial dimensions of the pulp, more precisely the x- and y-dimensions, were scaled by a factor of 0.5. While this does not universally reflect anatomical reality, the height, i.e., the z-dimension, was left unchanged to ensure better comparability with the original model, as the primary objective was to investigate the effect of varying pulp thickness along the direction of illumination. Examining the simulation results for a blood concentration of 5.0% in the reduced pulp (see Figure 3b), the same general trends and spectral features observed in the original model (Figure 3a) can be identified, though with altered magnitudes. Once again, in the HbO_2_ case, two distinct dips between 500 nm and 600 nm are observed, while these features disappear in the Hb case. However, due to the reduced pulp volume and consequently shorter photon paths through the pulp, the difference between the transmission spectra for different oxygen saturations is noticeably diminished. This is also reflected in the pulp path fraction, where $s_{p}$ is now less than or equal to $s_{g}$ for wavelengths above 450 nm. On the other hand, the reduced pulp volume results in smaller drops in the pulp path fraction in the 400–450 nm range for both HbO_2_ and Hb cases, as ’direct’ detection paths are no longer as strongly blocked by the pulp. For the pus-filled pulp, the characteristic HbO_2_ double dip caused by gingival absorption is again present, along with the two distinct absorption peaks of pus at approximately 475 nm and 640 nm, albeit with reduced prominence. For the lower blood concentration of 0.5% (see Figure 3d), with a reduced pulp size, once more, similar effects are observed as in the corresponding original model in Figure 3c. Again, the shrinkage of the pulp cavity leads to a pronounced convergence of the transmission spectra for SO_2_ =0% and SO_2_ =100%, with both spectra exhibiting lower intensity from approximately 450 nm onward compared to the original model. Although this may not seem intuitive at first glance, it can be explained by the concurrent increase in dentin volume as the pulp volume is reduced. Comparing the assumed dentin absorption with blood absorption (see Figure 2b) reveals that dentin exhibits a higher absorption coefficient in this wavelength range, resulting in the observed intensity reduction. The same effect is also evident when comparing Figure 3a and Figure 3b; however, due to the higher blood concentration in the pulp, the intensity reduction occurs only beyond approximately 600 nm.

### 3.2. Spectral Transmission of the Molar Model

**Figure 5:**
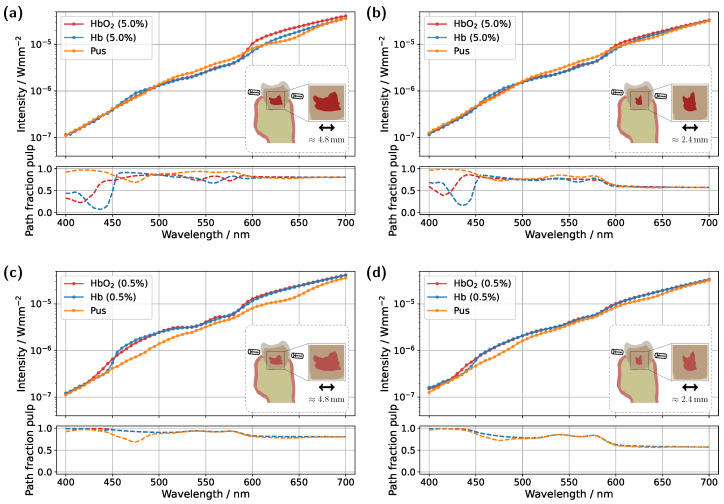
Simulated transmission spectra for the molar model. (**a**) Unmodified tooth model with a blood concentration of 5.0% in the pulp. (**b**) Tooth model with a reduced pulp volume and a blood concentration of 5.0%. (**c**) Unmodified tooth model with a blood concentration of 0.5% in the pulp. (**d**) Tooth model with a reduced pulp volume and a blood concentration of 0.5%. The insets display vertical cross-sections of the tooth model, highlighting the maximum pulp extension along the section plane. The respective subplots illustrate the pulp path fraction $s_{p}/\left(s_{p}+s_{g}\right)$ as a function of wavelength.

Analogous to the previously analyzed incisor model, the same simulation cases were then examined for a molar tooth (see Figure 5). As before, light was introduced and detected along an axis corresponding to the intersection of the sectional planes shown in Figure 1b, just above the gingiva. However, compared to the incisor model, the significantly larger dimensions of the molar result in a considerably longer optical path to the detector. In particular, the dentin and pulp tissue are substantially wider along the propagation direction, leading to expected differences in the transmission signal, primarily a reduction in intensity due to the increased optical attenuation. This expectation is confirmed in the simulation case with a 5.0% blood concentration (see Figure 5a).

**Figure 6:**
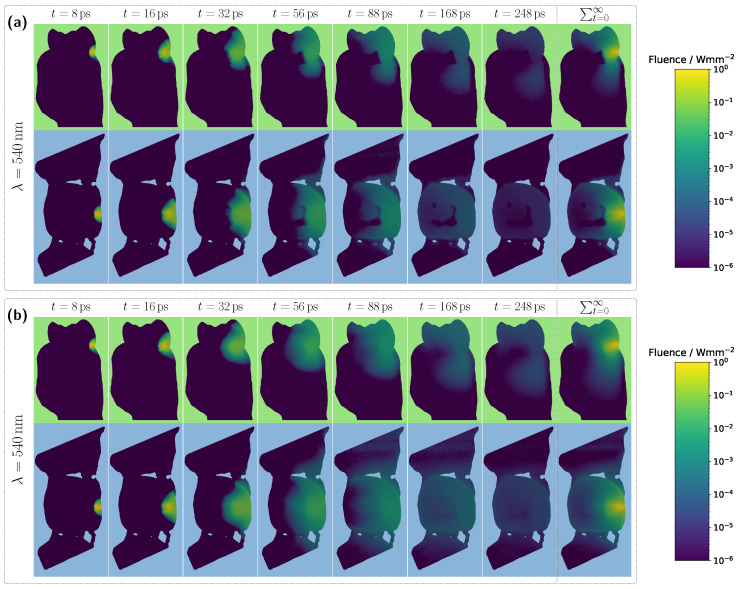
Time-resolved evolution of fluence for the unmodified molar model with a pulp filled with deoxyhemoglobin at a blood concentration of 5.0%. (**a**) Incident wavelength of 435 nm. (**b**) Incident wavelength of 540 nm. The top and bottom rows show the vertical and horizontal cross-sections of the tooth model, respectively. The rightmost column in each case represents the steady-state distribution of fluence.

Overall, the detected signal is approximately an order of magnitude lower compared to the corresponding incisor spectrum in Figure 3a. Another major difference concerns the shape of the transmission spectra for the different pulp fillings. In this case, the spectra for all three scenarios are much closer together, with only minimal traces of the characteristic HbO_2_ double dips and significantly weaker spectral features of pus absorption, where only the peak at approximately 640 nm remains slightly visible. These observations can be attributed to the pulp geometry. Firstly, the cross-sectional area of the pulp is significantly larger than in the incisor model. Secondly, the axis of light propagation and detection passes only marginally through the pulp tissue, meaning that most of the detected light must first be scattered a lot of times within the dentin before reaching the pulp. Additionally, the larger pulp cross-section blocks light more effectively. The combination of these effects results in a reduced influence of pulp composition on the transmission signal, manifesting as spectral curve convergence and weakened spectral features. For longer wavelengths, where attenuation within the pulp decreases, larger spectral differences between the different pulp fillings become evident beyond approximately 590 nm. In particular, for differentiating between HbO_2_- and Hb-filled pulp, the significantly different slopes of the spectra, attributable to their respective absorption profiles (see Figure 2), allow for improved distinction. In terms of diagnostic application, for the molar model under consideration, reliable differentiation would likely only be feasible when including this spectral range beyond 590 nm. Examining the pulp path fraction in the subplot of Figure 5a, absorption dips are once again observed for blood-filled pulps in the 400–450 nm range, though these are even more pronounced than in the incisor model due to the larger pulp dimensions. As previously shown, high absorption in this spectral range causes the pulp to block incident light almost entirely, resulting in most detected photons having taken longer paths through the gingiva rather than the pulp. This effect is further confirmed in the internal light distribution, see Figure 6a.

For an assumed Hb-filled pulp at 435 nm, incident light cannot pass directly through the pulp and must instead propagate around the pulp cavity before reaching the detector. Conversely, at a higher wavelength of 540 nm (see Figure 6b), a larger proportion of light can take a more direct path to the detector, resulting in an increased pulp path fraction. Regarding the pulp path fraction, a key distinction between the molar and incisor models becomes evident. In the incisor model, from approximately 450 nm onward, all cases exhibited nearly identical path fractions, with spectral features primarily influenced by gingival HbO_2_ absorption. In contrast, for the molar, the larger pulp cavity leads to distinctly different spectral curves for each simulated case, with the absorption characteristics of the respective pulp filling determining the final signal shape. Furthermore, for blood-filled pulps (see Figure 5a), similar pulp path fractions are reached beyond 450 nm, as in the incisor model, aside from variations caused by pulp absorption. This suggests that although larger pulp volumes generally lead to longer photon paths through the pulp, the resulting increase in path length is counterbalanced by greater attenuation along these extended trajectories.

Moving on to the model with a blood concentration of 0.5% (see Figure 5c), it becomes evident that in the spectral range between 500 nm and 600 nm, the spectral characteristics of the blood-filled cases become more pronounced, while the path fractions converge across most of the visible wavelength range. Additionally, the overall detected intensity increases. These three effects can be attributed to the reduced optical attenuation along the pulp. The incident light paths can now pass through the pulp more freely, experiencing less attenuation, resulting in a smaller contribution from photons that have traversed the gingiva. This is also reflected in the overall increase in the pulp path fraction across the spectral range. Nevertheless, the fact that the transmission spectra of both blood-filled cases nearly overlap suggests that the gingiva could still significantly influence the signal shape. This does not contradict the previous findings, as the HbO_2_/Hb absorption in the pulp is now approximately an order of magnitude lower than the gingival oxyhemoglobin absorption. Consequently, even in this case, the influence of the gingiva could lead to a misinterpretation in diagnosis.

In the modified model with a blood concentration of 5.0% and a reduced pulp size (Figure 5b), where the radial dimensions in the x- and y-directions were scaled by a factor of 0.5, analogous to the incisor model, similar signal characteristics and features as in the original model (Figure 5a) are observed, albeit with altered magnitudes. The most significant difference arises in the spectral range beyond approximately 590 nm. Due to the smaller pulp size and its consequently diminished influence on the transmission spectrum, the previously observed differences between the various pulp fillings are further reduced, making potential diagnostic differentiation even more challenging. Additionally, a lower signal intensity is observed compared to the original model. This effect can be explained analogously to the previously analyzed incisor model. The reduction in pulp size is accompanied by an increase in dentin volume. Given the assumed optical properties, the increased dentin volume leads to a further decrease in signal intensity, as dentin absorption becomes more dominant in this spectral range. Furthermore, in the pulp path fraction, the minima of both blood-filled cases below 450 nm and the pus-filled case at approximately 475 nm are reduced. Additionally, across the remaining wavelength range, the curves further converge and exhibit an overall decrease in magnitude. Both effects can again be attributed to the reduced influence of the pulp due to its smaller volume. A similar trend is also observed in the model with a blood concentration of 0.5% and a reduced pulp size (see Figure 5d), but in this case, the effects become apparent already at shorter wavelengths. This is due to the lower blood concentration, which reduces blood absorption, causing the transmission characteristics to shift accordingly.

Overall, the results presented in Figure 5 suggest that, when aiming to sensitively detect (blood) absorption differences within the pulp, the optimal spectral region depends on the absolute level of pulp absorption. In cases of high absolute pulp absorption, as shown in Figure 5a, the most suitable region is where blood absorption is relatively low, but the difference between oxy- and deoxyhemoglobin is large, such as around 600 nm or 470 nm. Conversely, in cases of low absolute pulp absorption, as in Figure 5d, the most informative region lies where blood absorption is high and the spectral differences between oxy- and deoxyhemoglobin are also significant, for example, around 435 nm. This means that the ‘optical absorption thickness’, i.e., the product of the absorption coefficient and the mean path length through the pulp, should, as a general rule of thumb, lie approximately around a value of 1, such that a change in absorption results in a significant change in transmission.

### 3.3. Strategies for Minimizing Gingival Contributions

**Figure 7:**
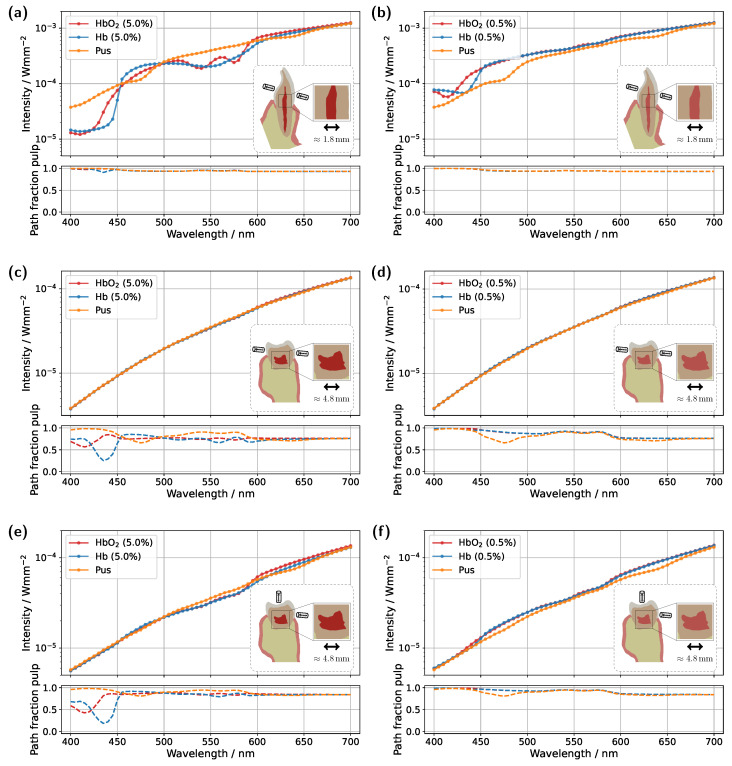
Transmission spectra for the incisor and molar models under variation of the illumination and detection axes. (**a**) Incisor model with a blood concentration of 5.0%. (**b**) Incisor model with a blood concentration of 0.5%. (**c**,**e**) Molar model with a blood concentration of 5.0%. (**d**,**f**) Molar model with a blood concentration of 0.5%. In (**a**–**d**), the illumination and detection axes are positioned approximately 2 mm above the gingival margin, whereas in (**e**,**f**), light is introduced just above the gingiva and the detector is placed approximately orthogonal to the illumination axis on the occlusal surface. The insets show vertical cross-sections of the tooth model, indicating the maximum pulp extension along the respective section plane. The lower subplots display the pulp path fraction $s_{p}/\left(s_{p}+s_{g}\right)$ as a function of wavelength.

Having demonstrated that a naive approach to using transmission spectra for assessing pulp vitality can easily lead to misinterpretations, two strategies will now be explored to potentially make this methodology less susceptible to errors. Specifically, the focus will be on whether the influence of gingival HbO_2_ absorption can be partially or even completely suppressed. As a first intuitive approach, the position of the illumination and detection axes was adjusted, see Figure 7, with the detector and source placed approximately 2 mm above the gingival margin. This adjustment was made in the hope that the increased distance would result in reduced contributions from the gingiva.

For the incisor model with a blood concentration of 5.0%, the intended effect is clearly reflected in the pulp path fraction (see subplot in Figure 7a), which now exceeds 0.9 across almost the entire spectral range. Compared to the original illumination and detector position (Figure 3a), a significantly smaller portion of the detected photon paths now traverses the gingiva, as expected. This is also evident in the transmission spectra, where the characteristic double dip caused by gingival oxyhemoglobin absorption is no longer present in the pus case. At a blood concentration of 0.5% (see Figure 7b), the double dip between 500 nm and 600 nm is also no longer observed in either the Hb or pus case, although it was clearly present in the original setup (Figure 3c). Furthermore, the spectra of the different pulp fillings are now even more closely aligned, as the influence of the pulp decreases further due to its significantly narrower cross-section, now more than half its original width, along the illumination axis. Nevertheless, due to the favorable optical absorption thickness, a pronounced difference between the two blood-filled cases is observed around 435 nm. In the case of the molar model with a blood concentration of 5.0%, the adjustment of the illumination and detection axes unfortunately did not produce the desired effect (Figure 7c). Here, the transmission spectra for the different pulp fillings are nearly identical, indicating that the pulp has little to no influence on the recorded signal. This becomes understandable when examining the 3D geometry in Figure 1c. The shifted axis causes direct photon paths to bypass the pulp tissue entirely, passing instead between the pulp horns. Unsurprisingly, the same outcome is observed at a blood concentration of 0.5%, making it evident that, under this configuration, a diagnostically meaningful classification of pulp vitality would not be feasible. Therefore, an additional setup was examined for the molar model (see Figure 7e,f), in which the light source was positioned as in the original arrangement (Figure 5), while the detector was placed nearly orthogonally to the illumination axis on the occlusal surface. At a blood concentration of 5.0% (Figure 7e), a distinguishable difference between the various pulp fillings is once again apparent, and the pulp path fraction is significantly increased compared to the original setup in Figure 5a. This indicates that the gingival contribution was effectively reduced using this configuration. A similar effect is observed at a blood concentration of 0.5%, although in this case, due to the lower blood absorption in the pulp, the spectra lie closer together. The only drawback of this measurement geometry is that, in contrast to the incisor model, it does not allow for a straightforward differentiation between the two blood concentrations based on the prominence of the oxyhemoglobin double dip. However, utilizing wavelengths of around 435 nm and 590 nm may offer a viable solution, due to the optimal optical absorption thickness in these regions. Furthermore, pus should be clearly detectable in both Figure 7e and Figure 7f. The authors further note that an additional measurement geometry was investigated for the molar, in which the illumination and detection axes, intended as an improvement over the setup in Figure 7c,d, were directed toward the left pulp horns (see Figure 1c). In this case, however, due to the proximity to the lateral gingival tissue and the stronger optical attenuation along the pulp, increased contributions from the gingiva were observed, with a mean path fraction of approximately 0.6. Apart from that, such precise positioning of the illumination and detection axis would require an additional imaging modality.

**Figure 8:**
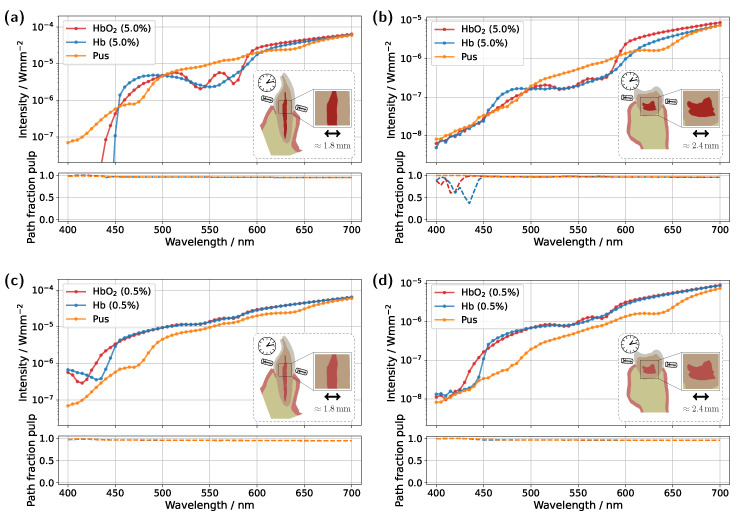
Time-gated transmission spectra for the incisor and molar model. (**a**) Unmodified incisor model with a blood concentration of 5.0% in the pulp. (**b**) Unmodified molar model with a blood concentration of 5.0% in the pulp. (**c**) Unmodified incisor model with a blood concentration of 0.5% in the pulp. (**d**) Unmodified molar model with a blood concentration of 0.5% in the pulp. The insets display vertical cross-sections of the tooth model, highlighting the maximum pulp extension along the section plane. The respective subplots illustrate the pulp path fraction $s_{p}/\left(s_{p}+s_{g}\right)$ as a function of wavelength.

As a second approach, the application of a time-gated detector was investigated. In this method, detection occurs within a defined time window to primarily register direct photon paths that have predominantly interacted with the pulp. Theoretically, this approach filters out longer photon paths, which have a higher probability of interacting with the gingiva, thereby reducing their influence on the detected signal. Considering statistical limitations, a tolerance was introduced in the integration time. This ensures that not only the very first detected photons are recorded but also those arriving approximately 15 ps (incisor) and 60 ps (molar) later. Figure 8 presents the time-gated transmission spectra for the original incisor and molar models at blood concentrations of 5.0% and 0.5%, respectively. As before, pulp fillings with oxyhemoglobin, deoxyhemoglobin, and pus were examined.

Starting with the incisor model at a blood concentration of 5.0% (see Figure 8a), the characteristic HbO_2_ double dip between 500 nm and 600 nm is once again clearly visible, while in the Hb case, it disappears, and the transmission spectrum follows the spectral characteristics of deoxyhemoglobin absorption. In the pus case, the HbO_2_ double dip that was still visible in the steady-state simulation (Figure 3a), due to gingival absorption, is now suppressed by time-gating. However, it remains faintly detectable, as the chosen tolerance in time-gating still allows minor contributions from the more strongly absorbing gingiva. This is confirmed by the pulp path fraction of approximately 0.95. Examining the same incisor geometry with a reduced blood concentration of 0.5% (see Figure 8c), the associated decrease in pulp absorption again leads to a convergence of the HbO_2_ and Hb spectra. However, compared to Figure 3c, no distinctly pronounced oxyhemoglobin double dip is observed in the Hb case, even though both curves, HbO_2_ and Hb, exhibit a similar spectral shape. As previously seen in the pus case, it is likely that the curves are still slightly influenced by the much higher gingival absorption. To further reduce this effect, a combination with the previous approach, positioning the detector further from the gingiva margin, could be beneficial. It is also worth noting that fine-tuning of the time-gating could theoretically reduce the gingival influence to an arbitrary degree. Turning to the molar model with a blood concentration of 5.0% (see Figure 8b), a substantial improvement in the differentiation of the different pulp fillings is observed compared to the steady-state simulation in Figure 5a. Within the spectral range between 500 nm and 600 nm, the HbO_2_ double dip is now more pronounced, while, as expected, it is absent in the Hb case. Across the remaining wavelength range, larger differences between the various transmission spectra are evident, which could potentially improve diagnostic differentiation. For a blood concentration of 0.5% (see Figure 8d), a noticeable improvement compared to Figure 5c is also observed. Once again, time-gating enhances the prominence of the HbO_2_ double dips, and the differences between the curves are more pronounced throughout the spectrum, leading to improved differentiation between the different pulp fillings. In particular, the blood-filled cases again show pronounced differences around 435 nm, attributable to the small amount of blood present in the pulp. Additionally, in the pus case, the absorption characteristics become more distinct in both Figure 8b and Figure 8d. The absorption peaks at approximately 475 nm and 640 nm are now clearly visible in the transmission spectrum.

## 4. Conclusions

In this study, a digital optical twin of an incisor and molar model was developed based on the MC method to investigate the dynamics of various model components and enhance the understanding of light propagation within the tooth. This, in turn, aims to contribute to the potential improvement of pulp vitality assessment in optical diagnostic procedures.

The simulations clearly demonstrate that the spectral analysis of transmission signals, while seemingly a promising tool for assessing pulp vitality, carries inherent risks of misinterpretation. In the incisor model, it becomes evident that peripheral tissues, such as the gingiva, can significantly influence the detected transmission spectrum. In particular, blood absorption in the gingiva can overshadow spectral characteristics, leading to a misleading appearance of oxygenated blood in the pulp, even in cases of a deoxygenated or pus-filled pulp. This effect is further exacerbated as the pulp volume decreases. Overall, these findings theoretically confirm, as previously observed experimentally [11], that a reliable diagnosis cannot be ensured without additional adjustments. As a side note, to the best of our knowledge, the optical detection of pus within the dental pulp has been investigated here for the first time. Diagnostic challenges also emerge in the molar model, though for different reasons. Due to the overall larger size of the tooth and the pulp itself, spectral characteristics of the pulp filling in vital cases are significantly less pronounced compared to the incisor. Here, differentiation is only feasible when incorporating wavelengths above 590 nm. However, as blood concentration decreases and the vitality status of the pulp declines, spectral features such as blood absorption become more discernible due to reduced attenuation. Nevertheless, the signal remains influenced by the gingiva. Since this observation contradicts intuitive expectations, it could also contribute to diagnostic errors.

To enhance the spectral characteristics of the pulp filling while simultaneously suppressing the influence of the gingiva, two strategies were investigated. The first, intuitive approach involved shifting the illumination and detection axis approximately 2 mm above the gingival margin to counteract the effects of gingival blood absorption. For the incisor model, this adjustment resulted in a significant improvement, effectively suppressing most contributions from the gingiva. However, in the molar model, the pulp had almost no detectable influence on the transmission signal. This was attributed to the pulp position and geometry in the examined tooth model, as the increased distance from the photon paths rendered pulp contributions negligible. To address this, an additional modification was introduced for the molar. The illumination and detection axes were arranged approximately orthogonally, with the detector positioned on the occlusal surface of the tooth. This configuration successfully suppressed a substantial portion of gingival contributions, even in the case of the molar. As a second approach, a time-gated detector setup was examined. By selecting an optimal integration time, ‘direct’ photon paths, those primarily interacting with the pulp, could be reliably isolated, ensuring that all expected spectral features were detected in both the incisor and molar models. However, a drawback of this method lies in the selection of the time gate, as this parameter critically influences the extent to which residual contributions from the gingiva are included in the signal. Nevertheless, simulations could be used to determine the optimal gating window.

Overall, it was demonstrated that tooth and pulp geometry, oxygen saturation and concentration, gingival blood absorption, and the material composition of the pulp can significantly influence the transmission spectrum. Effectively filtering out gingival interference requires specific adjustments, such as shifting the illumination and detection axes and/or implementing time-gating. Furthermore, the results illustrate that the choice of wavelength, and thus the selection of an optimal optical absorption thickness, is crucial for sensitively detecting changes in pulpal absorption. The key findings of this study highlight the necessity of first understanding light propagation within the tooth, such as through digital optical twins, to ensure high diagnostic quality in optical diagnostic techniques. Future investigations and extensions of the simulation model aim to incorporate more realistic tubule orientations, e.g., based on cross-sectional imaging including spatially resolved density or age-related reductions in tubule radius [41], as well as to consider a broader range of tooth models to cover non-ideal conditions such as the presence of caries, restorations, or root canal treatments.

## Data Availability

The raw data supporting the conclusions of this article will be made available by the authors on request.

## Appendix A. Impact of Optical Property Variability

In the following, the influence of variations in optical properties, such as those arising from measurement uncertainties or biological variability, will be demonstrated using the original model of the incisor tooth with a pulp filled with oxyhemoglobin. For this purpose, Figure A1 presents, for various model components, a comparison of the transmission signal obtained from simulations using the original optical properties and those in which the properties were varied by ±50%.

Starting with the peripheral tissues—specifically bone and gingiva (Figure A1a,b)—only a low sensitivity to changes in scattering is observed, resulting in no significant impact on the transmission spectrum. In contrast, variations in the tubule area number density and radius (Figure A1c,d) show a more pronounced influence, particularly at wavelengths below 450 nm, where a noticeable shift in the transmission curves occurs. The largest quantitative difference, however, can be observed when altering the reduced scattering coefficient of the dentin matrix (see Figure A1e). While changes in the pulp’s $\mu_{s}^{\prime }$ (Figure A1f) also lead to observable differences in the detected signal, their magnitude remains lower compared to those induced by changes in the dentin matrix. In all cases considered, it is confirmed that while variation in optical properties can lead to quantitative differences in the transmission signal, the spectral characteristics, such as the two HbO_2_ absorption dips, remain preserved. Notably, the pulp path fractions shown in Figure A1a–f also indicate that the influence of the gingiva is, at most, minimally affected. Thus, the core findings and interpretations of the previous simulations remain valid, despite uncertainties in the optical properties. A quantitative overview of the relative deviations in Figure A1 with respect to the original simulation parameters can be found in Table A1.

**Table A1:** Quantitative analysis of the relative deviations in the transmission spectra under variation of the scattering properties of different model components. The calculated mean, minimum, and maximum refer to the absolute values of the relative deviations.

Parameter Variation		Mean	Minimum	Maximum	@540 nm	@575 nm
Bone $\mu_{s}^{\prime }$	+50%	2.9	+0.3%	+12.3%	+0.5%	−2.4%
	−50%	1.6	+0.2%	+5.9%	+0.3%	−1.4%
Gingiva $\mu_{s}^{\prime }$	+50%	6.7	−1.0%	+14.8%	−4.2%	−6.7%
	−50%	3.8	+0.1%	+7.2%	+4.6%	+2.1%
Tubules density	+50%	33.9	+7.6%	+137.4%	+37.4%	+29.6%
	−50%	13.0	−5.3%	−35.9%	−14.1%	−15.0%
Tubules radius	+50%	45.3	+10.1%	+180.3%	+52.0%	+42.6%
	−50%	14.9	−6.6%	−40.7%	−15.8%	−18.4%
Dentin matrix $\mu_{s}^{\prime }$	+50%	76.1	+32.3%	+107.7%	+76.3%	+67.8%
	−50%	34.7	−9.9%	−44.2%	−33.8%	−34.0%
Pulp $\mu_{s}^{\prime }$	+50%	33.1	−2.3%	+55.5%	+45.0%	+38.8%
	−50%	19.1	−0.2%	−29.5%	−22.9%	−25.0%
